# Perceived causes of cancer in a rural community of Ethiopia: a qualitative study

**DOI:** 10.1080/16549716.2024.2401862

**Published:** 2024-09-17

**Authors:** Abigiya Wondimagegnehu, Muluken Gizaw, Lidya Genene Abebe, Brhanu Teka, Andreas M. Kaufmann, Tamrat Abebe, Shannon A. McMahon, Adamu Addissie, Eva J. Kantelhardt

**Affiliations:** a Department of Epidemiology and Biostatistics, School of Public Health, College of Health Sciences, Addis Ababa University, Addis Ababa, Ethiopia; b Global Health Working Group, Institute of Medical Epidemiology, Biometrics and Informatics, Martin-Luther-University, Halle-Wittenberg, Germany; c NCD Working Group, School of Public Health, Addis Ababa University, Addis Ababa, Ethiopia; d Department of Microbiology, Immunology and Parasitology, School of Medicine, College of Health Sciences, Addis Ababa University, Addis Ababa, Ethiopia; e Clinic for Gynecology, Charité-Universitätsmedizin Berlin, Corporate Member of Freie Universität Berlin, Humboldt-Universität zu Berlin, and Berlin Institutes of Health, Berlin, Germany; f Heidelberg Institute of Global Health, Heidelberg University Hospital, Heidelberg, Germany; g Department of International Health, Johns Hopkins Bloomberg School of Public Health, Baltimore, USA; h Department of Gynaecology, Martin-Luther-University, Halle-Wittenberg, Germany

**Keywords:** Perceptions about cancer, rural community, misconception of cancer causes, health education, culturally tailored intervention

## Abstract

**Background:**

Although cancer incidence and mortality are rising in Ethiopia, lay and health professional perceptions of the disease remain limited.

**Objective:**

To explore perceptions of cancer, including its causes, signs and symptoms, and transmission within a rural community in Ethiopia.

**Methods:**

We conducted a qualitative study in four rural neighbourhoods of Butajira in central Ethiopia. Seven Focus Group Discussions (FGDs) and six In-Depth Interviews (IDIs) were held with community members, women representatives, religious leaders and key informants using two interview guides (divided by method). Behaviour change theories and a community research framework were used to summarize the findings.

**Results:**

Across respondent categories and data collection methods, respondents described cancer or nekersa, which translates as ‘an illness that cannot be cured’, as serious and fatal. Cancer was further viewed as becoming more common and as underpinning more deaths particularly among women. Causes of cancer largely focused on individual behaviours namely mitch (referring to exposure to sunlight), poor personal hygiene and urinating on the ground/dirty areas. Almost all participants strongly related cancer to a wound that does not heal and entails a foul-smelling discharge. Bleeding and weight loss were other commonly mentioned complaints of cancer.

**Conclusions:**

Although cancer is known among rural communities in this area, misconceptions about cancer aetiology and conflation of the signs and symptoms of cancer versus other diseases merit health messaging. Our study calls for design research to determine how to culturally tailor educational materials and deliver health campaigns regarding cancer causes, signs and symptoms within this context.

## Paper context

- **Main findings:** Despite efforts to enhance cancer awareness and understanding among rural residents in Ethiopia, various misconceptions persist regarding its causes, transmission and prevention measures.
- **Added knowledge:** Unfavourable community perceptions, such as viewing cancer as a death sentence and believing in scientifically unfounded causes and modes of transmission, can significantly impede efforts for cancer prevention and control.
- **Global health impact for policy and action:** Effective global health action and policy must start from the local understanding of the terminologies and the perceived cause of the disease to design all-inclusive cancer prevention and control strategies.

## Background

Cancer incidence and mortality are substantially rising worldwide [1]. An estimated 19.2 million incident cases were reported around the world, and there are projected to be 30.2 million over the next two decades [1,2]. Of these, two-thirds of new cancer diagnoses will occur in low- and middle-income countries (LMICs) [3]. In 2020, nearly 10 million people died due to cancer, with a majority of reported deaths in Asia (58.1%), Europe (19.6%) and Africa (7.1%) [1,2].

In Ethiopia, an estimated 77,352 new cancer cases and over 51,865 related deaths were reported in 2020 [1]. Consistent with other LMICs, cancers of the breast and cervix were the two most common cancers in Ethiopia [4–6] accounting for 20.9% and 9.8% of cases, respectively [1].

Although diagnosis of cancer is increasing in Ethiopia, knowledge about cancer including risk factors and clinical manifestations of malignant tumours remains low within the general public but also in formal clinical settings. According to a study conducted among medical and health science students in Gondor University, knowledge of breast cancer and self-breast examination was very low [7]. In a study among general students in Debre Berhan University, roughly a third (195, 35.6%) reported having good knowledge of cervical cancer [8]. Health extension workers also reported limited knowledge about cervical cancer [9], suggesting that the situation might be worse within rural communities where a majority of people not only lack access to basic health infrastructure but also are limited in terms of education that could enhance health literacy [10]. Community-based studies conducted in the southern part of the country identified that the knowledge of cancer is as low as 11% [11,12], but this varied by place of residence, with rural dwellers having relatively lower knowledge towards cancer in general.

Although some studies highlight that the word ‘cancer’ is familiar among study participants, many in Ethiopia have never heard the word [13] and comprehensive knowledge about cancer remains poor [7,8,14–16]. Cancer patients in Ethiopia often present at advanced stages of the disease [5,6] and usually seek treatment from traditional healers and religious leaders [17,18].

Several studies to date in Ethiopia have focused on quantitative insights regarding risk factors associated with cancer and community awareness in general [7–9,11,12]. Iterative exploration of lay perceptions towards cancer causes, local names, perceptions of signs and symptoms, mode of transmission and treatment options remains scarce. Furthermore, studies that do exist have focused largely on cervical and breast cancer screening rather than on a more holistic picture of cancer in general. The objective of this study is therefore to qualitatively explore local perceptions of cancer, in a more holistic sense, within rural communities of Ethiopia.

## Methods

### Study design and place

A qualitative study design was used to explore lay perceptions of cancer in rural Butajira, which is located 135 km from the capital Addis Ababa, Ethiopia. The district has 10 *kebeles*/villages with an estimated population of 175,682. This study was conducted in four rural *kebeles*, namely, Shershera Bido, Mesrak Meskan, Bati Lejano and Butajira town.

### Study participants and sample size

A total of 58 participants (35 males and 23 females) were involved in this study; six were health professionals who have been serving the community for at least one year, and the remaining participants were community members (Table 1). Participants were purposively selected based on the duration of stay working in the health facility (minimum one year) and their role in the community. An attempt was made to achieve the maximum variation of participants by including both genders, different age groups, rural and urban residency, educational attainment, and role in the community.

**Table 1.** Characteristics of study participants, Butajira, Ethiopia.

Name of Village	No of IDI*	No of FGD^a^	Age range of participants	Male	Female	Role
Shershera Bido	2	2	25–37	9	9	1HEW^b^ & 1 MCH^c^ focal
Misrak Meskan	1	2	22–47	9	8	1 MCH focal
Bati Lejano	1	1	-^e^	8	1	1 HEW
Butajira town	2	2	28–50	9	5	2 MCH at the HC^d^ & Hospital
**Total**	**6**	**7**	**22–50**	**35**	**23**	–

^*-^IDI: In-Depth Interview, ^a^FGD: Focus Group Discussion, ^b^HEW: Health Extension Workers, ^c^MCH: Maternal and Child Health, ^d^HC: Health Centre, ^e^not recorded.

### Data collection tools and procedures

To facilitate FGDs and IDIs, two semi-structured interview guides were prepared based on the objectives of the study. The interview guides were initially prepared in English and translated into Amharic (the language employed in the region). The FGD and IDI guides entailed six main questions regarding how the community perceives cancer, cancer causes, signs and symptoms, mode of transmission, treatment and prognosis (see Appendix 1 & Appendix 2 for tools). The research team discussed interview guides and added a few more probing questions after conducting some IDIs. A total of seven FGDs with community elders, religious leaders, women’s representatives and *ekub/eder* (traditional saving and support system) leaders were conducted. FGDs lasted 75–105 minutes and included 6−9 participants each. To enhance privacy and encourage women to freely express their thoughts, separate FGDs were held between males and females in each kebele. In addition, six IDIs were conducted with key informants such as Maternal and Child Health (MCH) focal persons and Health Extension Workers (HEW) which lasted for about 22−37 minutes. Both the FGDs and IDIs were conducted by two data collectors with background in public health and who had experience facilitating qualitative data collection. We collected data until the point of saturation when there was no new idea emerging in either interviews or discussions. All interviews were audio recorded, and notes were taken during the interviews and discussions. The data were organized and appropriately labelled immediately after each session. Subsequently, all recorded IDIs and FGDs were transcribed and translated verbatim.

### Data analysis procedures

Data analysis was initiated concurrently with the data collection in order to modify the interview guide based on the newly emerging ideas and to decide on the level of saturation of the data. Daily debriefings were held with the entire research team along with two insiders who worked in the Butajira demographic surveillance sites and lived in the community for several years [19]. All emerging ideas and interpretations were discussed among the team members, and some additional probing questions were included to verify the findings. After repeatedly listening to the recorded audios, all of the transcriptions, field memos and debriefing notes were entered into QCA map software [20] for further analysis. All of the documents were coded line by line based on the objectives of the study and the main questions included in the interview guide. Subsequently, inductive content analysis was applied in order to summarize and explain the emerging categories and themes under each objective. After identifying emerging ideas corresponding to each objective, we incorporated behaviour change theories [21] and a community research framework [22,23] to succinctly summarize the main findings, given the diverse range of perceptions identified under each objective. Thus, we further categorized our findings into four areas: i) desirable and beneficial, ii) undesirable and harmful, iii) non-existent or incomplete and iv) benign perceptions. Desirable beneficial pertains to perceptions that are actively pursued within scientific knowledge and contribute to well-being or offer advantages to the community while undesirable harmful denotes perceptions that result in unfavourable outcomes, inflicting damage or adverse effects on the community. Non-existent or incomplete perception embraces those thoughts triflingly highlighted among the study participants that require introduction or further classification during intervention while benign perceptions are harmless misperceptions that need little attention. Data gathered from the community members and health professionals were triangulated through discussion with local personnel, and then, appropriate interpretations of the findings were organized into those different categories.

### Ethical clearance

Ethical clearance was obtained from the Research Ethics Committee of the School of Public Health and Institutional Review Board of the College of Health Sciences, Addis Ababa University with approval number 057/17/SPH. Oral informed consent was obtained from all participants, and confidentiality of the data was kept.

## Results

The results of this study are organized into three themes: a) general perception about cancer in the community b) perceived causes of cancer and c) perceptions towards signs/symptoms and transmissibility of cancer. Subsequently, we summarized these findings into four domains of the framework: desirable and beneficial, undesirable and harmful, non-existent or incomplete, and benign perceptions. Each theme and subtheme are described in detail below.

### General perception about cancer

Our study participants reported that cancer is a very dangerous disease and also expressed the seriousness of the diseases saying ‘deadly disease’, ‘dangerous and shocking disease’ and a disease which ‘stresses you out and cannot be easily treated’. For instance, a religious leader said the following, indicating that cancer diagnosis and treatment is a complicated process and requires an extended time and effort similar to a court process.

Nekersa (local name for cancer) is an incurable disease. A person who acquired cancer and who is accused in the court, will not be released easily! Even people curse someone saying may you acquire Nekersa! This means it is a kind of disease which will not be cured at all **(FGD: Men, Religious Leader)**

Most participants linked cancer to significant weight loss and said *‘We start recognising the changes in our body by observing our skin as it gets whiten and soften your bone. Then we will significantly lose weight.’* **(FGD: Women, Shershera Bido)**. Another male participant also expressed this as *‘Cancer eats your muscle, and you will significantly lose weight’* **(FGD: Men, Mesrak Meskan)**.

### Local names and meaning

The word ‘cancer’ is well known among the study participants, and it is reported that communities are becoming familiar with this word. However, the local name previously given for cancer was ‘*nekersa*’- meaning a disease which cannot be cured.

Nekersa is better to understand. Even our elderlies know the disease as nekersa. We recently start calling it cancer but from the beginning, we have been calling it nekersa. So, the well known word is nekersa **(FGD: Men, Shershera Bido)**

## Commonly known cancer types

Cancer of the breast and uterus are the most commonly known cancer types among the study participants. No respondent highlighted distinctions across endometrial, ovarian or cervical cancer. Additionally, respondents seldom mentioned cancer of the brain, neck, lung, abdominal organs, bone or leg. *‘Cancer usually affects the breast and cancer of the womb is the most common one’* **(FGD: Com. Rep, Kebele 04)**.

Some participants also reported that cancer can arise from different body parts and disseminate to the whole body: *‘Cancer can occur anywhere in our body’* **(FGD: Men, Shershera Bido)**, while another participant said: ‘*It may arise anywhere, and it doesn’t choose the place’* **(FGD: Men, Religious Leader)**.

Different perceptions reflected towards causes of cancer, signs/symptoms and transmission were categorized and explained as desirable beneficial, undesirable harmful, non-existent or incomplete with the last category benign presenting a target for change (Table 2).

**Table 2.** Cancer perceptions and opportunities for intervention in Butajira, Ethiopia.

	Desirable and Beneficial Perceptions	Undesirable and Harmful Perceptions	Nonexistent or Incomplete Perceptions	Benign Perceptions
Perceptions of Cancer	**Causes of cancer**– Unsafe abortion(s), multiple births, prolonged labour, and/or home delivery (contamination during delivery) cause cervical cancer– Accumulation of breast milk**Sign/symptoms**– Untreated wounds can be converted to cancer	**Causes of cancer**– Exposure to sunlight while breastfeeding**Sign/symptoms**– Considering bleeding as menstrual cycle– Confusing vaginal discharge with other STIs, haemorrhoids and fistula**Transmission**– Transmitted via sharing of sharp materials– Transmitted via breastfeeding	**Causes of cancer**– Having multiple sexual partners– Unprotected sex– Early marriage– Poor personal hygiene– Being pierced with sharp and rusted materials**Sign/symptoms**– Weight loss, swelling, cough, itching, vaginal discharge and burning sensation**Transmission**– Cervical cancer can be transmitted from female to male– Cervical cancer can be sexually transmitted similar to HIV and STIs.	**Causes of cancer**– Exposure to bad air (*Mitch*)– Exposure to sunlight after preparing local alcohols– Holding cents under the armpit– Wearing tight bras– Urinating on the ground/dirty areas– Urinating in the direction of the sun/moon.

## Perceived causes of cancer

Participants generally understand that cancer can be caused by bad air and call it by the local term *‘Mitch*’. In the community, there is a belief that if a woman gets exposed to sunlight while breastfeeding or if she gets out of her house sweating or after preparing some traditional alcohols, that woman may acquire breast cancer. The participants reported that this is how the victims themselves believe they got the disease.

They usually complain as they got the disease when they breast feed their children on sunlight and those women who made Areke (traditional alcohol) talk as they got the disease when they are exposed to bad air when they immediately come out of house after preparing it **(FGD: Com. Rep, Kebele 04)**

In addition, a few participants pointed out that holding cents under their armpit or around their breast during the hot season can cause cancer. *‘For breast cancer, its due to our small bag which we usually hold under our breast. So, if we are carrying a heavy bag during hot season, it can cause breast cancer’* **(FGD: Women, Shershera Bido)**.

For other types of cancers, such as leg, neck and skin cancer, it is assumed that untreated wounds will be converted to cancer over time. Not only community members but also health professionals describe this. A Health Extension Worker from Bati Lejano said *‘The other cause is if we didn’t properly treat wound, it might be changed to cancer through time. It’s related to diseases and wounds*’.

Poor personal hygiene, being pierced with sharp and rested materials, the accumulation of breast milk and wearing tight bras are also reported as other possible causes of cancer, while few respondents said that there are several factors and the actual cause of cancer is unknown. For example, one of the community representatives said that ‘*Cancer can be caused by several things’* and the MCH focal person from Butajira Health Centre stated *‘The causes are unknown. I can’t tell you as this is the exact cause of cancer’.*

Most women participating in this study reported that urinating on the ground/dirty areas, particularly in the direction of the sun/moon, can cause cancer. Even though they could not identify and specifically indicate the type of uterine cancer, they believe that this practice could cause cancer anywhere on the female reproductive organ, which is commonly called *‘Mahtsen’* in the local language meaning ‘womb’.

When we pee on the ground, the dirt will be inserted into our uterus and cause cancer. Similarly, if we pee on the side of the sun in a very hot day, that can also cause cancer and other several diseases **(FGD: Women, Shirshera Biro)**

In addition, they urinate on ashes. Then, this will be inserted into their uterus and cause cancer. Moreover, it’s not good to pee to the direction of the moon. If they pee to the moon, it will be like ‘Mich’ which has very burning sensation and will be converted to cancer later **(FGD: Men, Mesrak Meskan)**

As I usually observe, women pee on the ground while sitted. So, the dust particles will be inserted into their uterus and that will cause cancer. Umm … in addition, they don’t usually sit on clean areas. They don’t use underwear and just simply sit on the ground. So, this can easily expose them to dirties. Then it will be inserted into their uterus and cause cancer **(FGD: Men, Shershera Bido)**

In addition to the health professionals involved in this study, a few community members mentioned that having multiple sexual partners, unprotected sex and early marriage can be possible risk factors for cervical cancer. They also believe that cervical cancer can be transmitted sexually like other STIs and HIV.

Even though sexual intercourse is natural, having multiple sexual partners may cause cervical cancer. A woman may get the disease from one of her partners. **(FGD: Men, Religious Leader)**

*…* it’s due to having multiple sexual partners and early marriage can also transmit the disease **(FGD: Men, Mesrak Meskan)**

Furthermore, our results pointed out that the causes of cervical cancer are closely linked with poor personal hygiene and contamination during delivery. Particularly, home delivery, unsafe abortion, circumcision, prolonged labour and having multiple children are considered the main causes of cervical cancer in the community.

But the community perceives as this disease occurs when a woman give birth to many children and when she had prolonged labour **(IDI: MCH Head, Kebele 04)**

*…* during home delivery, women will be exposed to contaminations which finally leads to cervical cancer **(FGD: Men, Religious Leader)**

## Perceived signs and symptoms of cancer

The most commonly mentioned signs and symptoms of cancer by our study participants were open wounds. A majority of our respondents directly related the word cancer with wound and considered it the most common manifestation of the disease.

Itching sensations are another widely stated symptom of cancer among community members. Most of the time, burning and itching are considered an initial symptom of the disease which leads to a wound.

It usually starts by itching. First, he had an itching sensation on his toes. Then, the skin turned black and became like a hole between the two toes **(FGD: Men, Bati Lejano)**

Significant weight loss, bleeding, stabbing pain, swelling and cough are additional signs and symptoms of cancer listed by the participants. In contrast, regarding cervical cancer, the respondents described discharge and bleeding as the typical signs and symptoms of the disease. A 28-year-old housewife from Mesrak Meskan said *‘When we say cancer of the uterus, it usually has pus and discharge from the womb.’* Another woman from the same village stated *‘First, it will have some discharge which has awful smell. Then, it will be wounded’* **(FGD: Women, Mesrak Meskan)**

Bleeding is the other most frequently stated sign of cervical cancer. It is also indicated that women get confused with menstruation and do not immediately seek health care when they have vaginal bleeding. *“Women with cervical cancer have bleeding and usually relate it to the menstrual cycle, expecting it to be back to normal at some point”* **(IDI, MCH Head)**. Not only this, but many people also confuse cervical cancer with other diseases such as haemorrhoids, fistulas and STIs because of the similar signs and symptoms. For instance, a male participant from Bati Lejano said, *‘I don’t know whether this cancer (referring to cervical cancer) and haemorrhoids are similar or not’.*

## Perceptions towards the transmission of cancer

Our findings revealed that the community perceive that cancer can be transmitted from person to person through sharing sharp materials. For example, a religious leader said ‘*It might transmit from person to person through sharing sharp materials like razors, needles and nail cutters.’* **(FGD: Men, Religious Leader)**

In the community, it is also believed that cancer can be transmitted from person to person sexually, through direct skin contact with the wound and breastfeeding as well. Particularly for cervical cancer, some respondents mentioned that cancer can be transmitted due to bleeding during sexual intercourse: *‘There might be bleeding during sexual intercourse and the disease can be transmitted at that time’* **(FGD: Men, Mesrak Meskan)**. One respondent said that there is a possibility of transmitting the disease to males as well: *‘Maybe it’s transmitted from females to males through sexual intercourse*’ **(FGD: Women, Mesrak Meskan)**.

## Discussion

This study explored community perceptions of cancer, its perceived causes, common manifestations and the transmissibility of the disease. Our findings revealed that while all participants had heard of cancer, most lack information about its causes, signs and symptoms and mode of transmission.

Our participants reported that cancer is a deadly disease and has been given a local name, *‘Nekersa’*, which signifies its seriousness. A previous study performed in Jimma and Addis Ababa also reported that *Nekersa* is the local name given for cancer in the Amharic language to designate cancer as a serious and fatal disease that cannot be cured [24]. It is also mentioned that the word cancer is becoming very common nowadays, and people can easily understand what it means. This finding is similar to that of a study conducted in the Arsi zone which found out that the local name for cancer is ‘Kaansarii’ which is directly taken from ‘cancer’ and pronounced in Afaan Oromoo dialects [25]. Various studies in different parts of Africa also reported different local names given for cancer, namely ‘two remo’ in Uganda, meaning an illness that manifests with bleeding [26], ‘Mudzoko’ in Mozambique, which refers to a wound that does not heal [27], and ‘Umdlavuza’, a word conveys the internal mutation of disease in South Africa [28]. Understanding the etymological origins of the local names can help to design the right health education; also, for research purposes, we can more easily communicate with the general community using similar meanings and expressions.

One of the notable findings is that the majority of the study participants believe that exposure to the sun’s rays, ‘Mitch’, while sweating and after staying in the kitchen for a long time can cause breast cancer. Exposure to sunlight and the effects of the sun or heat exposure were commonly thought to cause different types of cancers. Similar perceptions were reflected in previous studies performed in Jimma, Addis Ababa and Hadya Zone [14,15,24]. Particularly, urinating in dirty areas when it was sunny or in the direction of the sun/moon and sitting on hot surfaces were mentioned as major causes of cancer of the uterus. A study undertaken in Dabat (Northern part of Ethiopia) also found similar perceptions towards the causes of cervical cancer, which is called ‘Mitat/Girefat’ in the local language. This means that ‘sudden exposure of the body to sunlight’ – particularly while menstruating, either urinating facing the sun, or water vapor, coming from the ground produced by sunlight enters the uterus during urination in an open space [15].

Few of the participants mentioned that if the wound persists for an extended period of time, the chance of being converted into cancer is very high. Similar thoughts were presented in a qualitative study in Uganda, which stated that the wound/lesion of STIs may gradually be converted into cervical cancer [26]. Poor personal/genital hygiene was the other commonly reported cause of cancer in this study which is in line with studies in coastal areas of Kenya [29], Uganda [30] and Southwestern Ethiopia [24].

Several misconceptions towards the causes of cancer were reported in previous studies. For instance, among the Navajo community (American natives), cancer is believed to be caused by lightening exposure and the consumption of contaminated food and water [31], and malnutrition, poorly cooked food and poor diet were mentioned among coastal Kenya [29], Ugandan [30,32] and Austrian cancer patients [33]. The ingestion of chemical agents and assaults by the spirits of the dead [30] were also other misconceptions towards the causes of cancer. Similarly, a few of our participants mentioned that holding coins under their armpit can be the possible cause of breast cancer. A similar misconception about the environmental risk factors of breast cancer was discussed among African American women who believed that deodorants containing aluminium, plastics, pesticides, and air and water pollution could be possible risk factors for breast cancer [34]. Nevertheless, chemicals and pesticides have biological plausibility; other misperceptions indicate that there are different speculations emerging in the community which might be due to a lack of information.

Concerning the signs and symptoms of cancer, a wound was one of the most commonly stated symptoms of cancer; also, almost all participants strongly related cancer to wounds that do not heal and have a foul smell discharge. A similar finding was reported from Kenya [30], the United States of America [31] and Mozambique [27]. Bleeding is the other frequently mentioned sign of cancer, particularly for cervical cancer. Similarly, a qualitative study conducted in Uganda found that the local name for cancer was even derived from this typical manifestation [32], and another study in the same country [35] and a previous study in Dabat Ethiopia [15] also revealed that pain, itching, burning, swelling, bleeding and foul-smelling discharge are the typical symptoms of cervical cancer. In contrast, the most commonly recognized symptoms of cancer in Malaysia were lumps/swelling which were reported in three quarters of the participants, and other symptoms such as cough, unexplained pain, weakness and weight loss were also reported by the respondents [36]. This variation might be due to differences in the study settings and design as our study was qualitative while the Malaysian study was a cross-sectional survey conducted by specifically listing scientifically proven cancer symptoms.

Weight loss was one of the prominent symptoms reported by our study participants. Similarly, a study in South Africa reported that many people perceive breast cancer as a physically and psychologically gruelling illness [28] and women with cervical cancer present with profound weakness, which the respondents attributed to low blood levels resulting from heavy vaginal bleeding, worries about death and poor feeding due to a loss of appetite [24]. As a result, participants in Mozambique stated that the wound inside the uterus will turn into pelvic pain and weakness and make one gains a ‘milky’ appearance [27].

Our findings suggest that vaginal bleeding and discharge are often confused with similar diseases such as haemorrhoids and STIs, with some women even being confused by their menstrual cycle. Various studies also reported similar findings and expressed a concern that due to a lack of knowledge about cervical cancer, relevant signs and symptoms can be missed [27,37]. Hence, this may contribute to delayed diagnoses and treatment seeking by considering it to be a natural phenomenon and something that can resolve itself or be easily treated by traditional medicine. This suggests that targeted health education in differentiating these symptoms and similar diseases can perhaps lead to earlier diagnosis.

Our study revealed that there is a belief in the community that cancer can be transmitted from person to person through different mechanisms. This is in line with a study performed in Gondor which stated that around 20% of medical and health sciences students believed that breast cancer can be transmitted from person to person [7]. In particular, uterine cancer is considered a sexually transmitted disease which might be related to the infectious nature of Human Papillomavirus which is the causative agent for cervical cancer. Similar thoughts were reflected in different qualitative studies in Uganda which found out that the community perceives that cervical cancer can be transmitted sexually and the male partner has the potential to acquire the disease and further spread it to other women [26,30,38]. Particularly, non-circumcised males were blamed for the transmission of cervical cancer among the Mozambique tribe [27].

The idea of contracting cervical cancer through sharp material was also indirectly reflected in another study in which the participants reported that the screening process by itself may expose the women to several diseases assuming that the next patient will be examined without disinfecting the screening materials [35]. Similar thoughts were also reflected in Kenya, which stated that being pierced by something sharp was considered a cause of breast cancer [29]. Not only among African populations but similar perceptions towards the transmission of cancer were also reflected in Navajo cancer survivors who were isolated from their family and the community due to the perspective of cancer being a contagion both in terms of physical contact and in verbal and spiritual aspects [31].

In general, some of the perceived causes of cervical cancer such as unsafe abortion, multiple births, prolonged labour and/or home delivery were categorized as desirable and beneficial as this may encourage the community to avoid those harmful practices. Similarly, the perception of the accumulation of milk causing breast cancer may encourage lactating women to express their breast frequently. In addition, untreated wound is perceived to cause cancer which in turn enhances health-seeking behaviour in the community. On the other hand, undesirable or harmful perceptions identified in this study need to be targeted for change as these thoughts may lead to stigma and discrimination against cancer patients, delay in presentation and discourage women from exposing their children to sunlight (vitamin D). Other different perceptions expressed with less depth and intensity merit introduction and classification while benign misconceptions require little attention in future awareness-creation programmes.

One of the strengths of this study is using both data collection techniques: IDIs and FGDs. We have included health professional’s opinions, which enabled us to triangulate our findings as they have been serving the community for a while and have a better understanding of the community’s perception towards cancer. In this study, there was at least one participant in each FGD who had either a close relative or neighbour who had been suffering from different types of cancer. Not excluding those participants might affect our result as this cannot be representative of the lay person perception since these participants have obtained relatively better information about cancer than the general population.

## Conclusion

Our findings indicate that cancer is perceived as the most dangerous and deadly disease, causing significant damage to both patients and families. Among the study participants, breast and uterine cancers were the most frequently mentioned types, consistent with common pathological diagnoses. Myths regarding cancer causes, such as exposure to sunlight ‘mitch’, urinating towards the moon/sunlight, untreated wounds and poor personal hygiene, were very common. These misconceptions can lead to self-blame, concealment of symptoms and psychological stress. To address these issues, it is crucial to educate opinion leaders to dispel myths, promote healthy living and relieve patient stress. Regarding signs and symptoms, most of the participants noted non-healing wounds, bleeding, vaginal discharge and significant weight loss as indicators of cancer. In addition, the community believes that cancer can be transmitted through sharing sharp materials, sexual contact, and breastfeeding. Specifically for cervical cancer, some mentioned transmission through bleeding during intercourse. This knowledge could be incorporated into teaching materials about the warning signs and transmissibility of cancer.

In general, our findings suggest that while cancer is known among rural communities, there is low awareness about its true causes, signs and symptoms, and transmission. Therefore, it is imperative to provide culturally tailored educational materials and campaigns about cancer for rural communities, specifically targeting and addressing undesirable and harmful perceptions. Concise and accurate information dissemination programmes, presented visually and in multiple formats with local terminology, can be highly effective. Involving traditional healers and religious leaders in advocacy can enhance trust in medical care and reduce the search for alternative treatments. Early diagnosis and screening modalities should be widely available, and it is crucial to have cancer survivors speaking out in rural communities to change the narrative of the certain death associated with cancer.

## Data Availability

The datasets used and/or analysed during the current study are available from the corresponding author upon reasonable request.

## Acronyms

FGD: Focus Group Discussion
GLOBOCAN: Global Cancer Observatory
HC: Health Centre
HEW: Health Extension Workers
HIV: Human Immunodeficiency Virus
HPV: Human Papilloma Virus
IDI: In-Depth Interview
LMICs: Low- and Middle-Income Countries
MCH: Maternal and Child Health
QCA: Qualitative Content Analysis
STIs: Sexually Transmitted Illness.

## Appendix 1. Tool for FGDs

### Socio-demographic characteristics of participants

**Table:**

**Variable**		**Response**	
**Participants code**	**Age**	**Sex**	**Role in the community**
P1			
P2			
P3			
P4			
P5			
P6			
P7			
P8			
**Place**		A. Shershera Bido C. Bati LejanoB. Mesrak Meskan D. Butajira town	
**Name of the data collector**		Moderator_______________________ Note taker______________________	
**Date of interview (DD/MM/YY)**		__________________	
**Time of interview**		Start:_________ End: _________	

1. What is cancer? Any local names given for cancer?

**Probing**: Please tell me about the types of cancer you heard about? List out the most common ones, who is most affected by cancer and what do you think of the reason?

2. What are the causes of cancer? How can cancer be caused?

**Probing**: For the different types,

3. What are the signs and symptoms of cancer?

**Pobing**: Duration, extent, differentiating features with other diseases

4. How can cancer be transmitted?

**Probing**: If they say transmittable, how?

5. How can cancer be prevented?

**Probing**: Before its occurrence, what can be done once diagnosed?

6. Please tell me about the types of treatment for cancer?

**Probing**: Medical and other alternative treatment options, its prognosis?

## Appendix 2. Tool for IDIs

### Socio-demographic characteristics of participants

**Table:**

**Variable**	**Response**
Age	________year
Sex	(A) Male (B) Female
Occupation/role in the community	
Place	Shershera Bido
	Mesrak
	Bati Lejano
	Butajira town
Name of the data collector	_________________________
Date of interview (DD/MM/YY	____________________
Time of interview	Start:_________ End: _________

1. How do the community understand cancer? Any local names given for cancer?

**Probing**: Please tell me about the types of cancer people know, who is most affected by cancer and what do you think of the reason?

2. How does the community perceive about causes of cancer? How do most people think about its causes?

**Probing**: For the different types, any misconceptions?

3. What are the most commonly mentioned signs and symptoms of cancer in the community?

**Pobing**: How do people differentiate from other similar diseases

4. How does the community perceive about transmission of cancer?

**Probing**: If they say transmittable, how? Please tell me about the perceived mode of transmission of cancer in the community?

5. How do the community members perceive about prevention, treatment and prognosis of cancer?

**Probing**: Perceived prevention methods, medical and other alternative treatment options considered effective in the community, its prognosis?
